# Candesartan prevents impairment of recall caused by repeated stress in rats

**DOI:** 10.1007/s00213-012-2829-3

**Published:** 2012-08-14

**Authors:** Jan Józef Braszko, Dominik Wincewicz, Piotr Jakubów

**Affiliations:** Department of Clinical Pharmacology, Medical University of Bialystok, Waszyngtona 15a, 15-274 Bialystok, Poland

**Keywords:** Stress, Memory, Angiotensin, Receptor, Antagonist, Avoidance, Recognition, Rat

## Abstract

**Rationale:**

Deleterious effects of psychological stress on memory are increasingly important. Overexpression of the AT_1_ angiotensin receptors in brain has been found to participate in several negative effects of chronic stress including hypertension and a cognitive impairment.

**Objective:**

In this study, we searched for the protective effects the AT_1_ angiotensin receptor blockade with candesartan against the adverse effects of repeated stress on recall of aversively and appetitively motivated behaviours in rats.

**Methods:**

Two groups of male Wistar rats were repeatedly stressed by keeping them daily (2 h/21 days) in tight plastic tubes. The subjects of the group 1 received candesartan (0.1 mg/kg, orally) each day before the stressing procedure. The rats of the group 2 received vehicle. Another two groups of rats (3 and 4) receiving candesartan and vehicle, respectively, were appropriately handled but not stressed. Next day, after ending the repeated stress procedure, all rats were tested in two cognitive paradigms: inhibitory avoidance (IA) and object recognition (OR).

**Results:**

Stressed animals displayed decreased recall of the IA behaviour (*p* < 0.01) and decreased OR (*p* < 0.05). These effects were not seen in the animals stressed and concomitantly treated with candesartan. The auxiliary tests designed to control for the possible unspecific contribution of motor (open field) and emotional (elevated “plus” maze) effects of the experimental procedures to results of the cognitive tests showed no such contribution.

**Conclusion:**

These data strongly suggest that the AT_1_ angiotensin receptor blockade effectively counteracts deleterious effects of stress on recall of aversively and appetitively motivated memories in rats.

## Introduction

Most of us experience stressful situations every day. These situations, perceived as a threat to our physical (e.g. traffic dangerous situation) or psychological (e.g. loss of job) integrity, evoke immediate adaptive mechanisms including activation of the hypothalamus–pituitary–adrenal (HPA), sympatho-adrenomedullary and sympatho-neural axes (de Quervain et al. 2009; Kvetnansky et al. 2009; Wolf 2009) initiated by signals from the limbic cortical areas such as medial prefrontal and cingulate cortex to paraventricular hypothalamic nucleus (PVN). The adaptive processes however become maladaptive and harmful to the organism once stressful stimuli occur repeatedly for a long time (weeks or months). Deleterious effects of chronic stress eventually manifest themselves in several pathologies such as gastric ulceration, hypertension, depression and a cognitive impairment (Buchanan et al. 2006; de Quervain et al. 1998; de Quervain et al. 2000; Saavedra 2005; Terfehr et al. 2011 ).

In the last few years, our laboratory has been particularly interested in the latter negative effect of stress in respect of its possible alleviation by simple and easy prevention by for example plant medicines such as the extracts from ginkgo biloba (Walesiuk et al. 2005, 2006) and St. John's wort (Trofimiuk and Braszko 2008; Trofimiuk et al. 2011) or dietary additive such as cod liver oil (Trofimiuk and Braszko 2011). Although mildly effective, these medicines do, by no means, sufficiently solve the problem of coping with stress-induced memory impairments.

In this context, recent discoveries by Saavedra's laboratory summarised in an excellent review (Saavedra et al. 2011) that candesartan and other AT_1_ angiotensin receptor blockers (ARBs) are extremely effective in the prevention or at least alleviation of a wide range of the stress-induced pathologies including gastric ulceration, adverse alterations of cerebrovascular structure and function in spontaneously hypertensive rats predisposing to brain ischemia and stroke and also brain inflammation appeared very promising. Importantly, part of the protective effects of ARBs was found to be unrelated to the inhibition of the angiotensin AT_1_ receptors and normalisation of blood pressure.

In rats, stress-induced overexpression of the AT_1_ angiotensin receptors in brain and periphery has been known for a long time (Aguilera et al. 1995a, b; Castren and Saavedra 1988; Jezova et al. 1998; Leong et al. 2002; Yang et al. 1996). Various types of stress, through peripheral sympathetic stimulation, increase renin activity and therefore production of circulating angiotensin II (Ang II) (Xang et al. 1993). The peptide, by stimulating AT_1_ receptors, contributes to the secretion of ACTH from the pituitary gland and subsequent release of mineralo- and glucocorticoids from adrenal zona glomerulosa and catecholamines from adrenal medulla (Ganong and Murakami 1987; Keller-Wood et al. 1986)*.* Stress increases Ang II content in many brain regions including the hypothlalamus (Xang et al. 1993). The resulting stimulation of the brain and pituitary Ang II systems, together with the increased AT_1_ receptor expression, activates the HPA axis and enhances corticotrophin-releasing hormone (CRH) formation and release (Aguilera et al. 1995a, b; Sumimoto et al. 1991) that further increases pituitary ACTH release followed by an increase of corticosterone formation and release (Ganong and Murakami 1987). The corticosterone in rodents (and cortisol in most other mammals) has been found to adversely affect retrieval of memory (for review, see Woodson et al. 2003; de Quervain et al. 2009).

In the present study, we attempted to counteract memory impairment produced by prolonged restraint stress in rats by simultaneously treating them with a low nonhypotensive dose of an AT_1_ angiotensin receptor inhibitor candesartan. The rationale for this approach was based on the considerable involvement of the Ang II AT_1_ receptor-mediated stimulation of HPA axis in the stress response. To produce memory deficits, we used daily 2-h restraint stress for 21 days (Magarinos et al. 1997; Walesiuk et al. 2005). Retrieval of memory of an aversively, motivated behaviour was measured using an inhibitory avoidance (IA) test (Ader et al. 1972). For comparison, we also measured retrieval of memory of an object in the object recognition (OR) test (Ennaceur and Meliani 1992) wherein memorising is motivated positively by natural curiosity.

To control for any unspecific contribution of the possible stress and /or candesartan-induced changes in the animals' motor performance to the results of our memory tests, we tested locomotor exploratory activity of rats in separate experimental and control groups. To control for the possible bias of the results of our cognitive tests by fear/anxiety resulted from the experimental procedure, we tested all rats in the elevated “plus” maze (Pellow et al. 1985).

## Methods and material

### Subjects

The experiments were conducted on male Wistar Cri:WI(Hannover) rats purchased from the Center for Experimental Medicine, Bialystok, Poland. This strain of rats is bred under special high standard nearly sterile conditions assuring their specific pathogen-free health status regularly checked according to the protocols provided by the Charles River Laboratories. They were 2 months old, weighing 140–160 g at the beginning of the study. The animals were then maintained in the temperature- (23 °C) and humidity- (50–60 %) controlled animal room in groups of five under constant 12-h/12-h light/dark cycle beginning at 0700 hours with free access to standard laboratory food and tap water. Principles of laboratory animal care according to the European Council Directive of 24 November 1986 (6/609/EEC) were observed in all procedures. All experiments were approved by the local Ethics Commission for Animal Experimentation.

### Procedures

#### Stress procedure and the drug treatment

Four groups or animals (*n* = 14–16) were used. Rats of the groups 1 and 2 underwent repeated restraint stress procedure described by Magarinos et al. (1997) with modifications (Walesiuk et al. 2005). Briefly, each rat to be stressed was daily placed in a restrainer (21 days/2 h) which was a tight transparent plastic tube 20-cm long and 7 cm in diameter. The restrainer was then closed with a Plexiglas lid. Both the restrainer and the lid were perforated for breathing. The animals fit tightly in the restrainers, and it was not possible for them to turn around. The subjects of group 1 received candesartan (candesartan cilexetil, AstraZeneca Södertälje, Sweden, 0.1 mg/kg, orally) suspended in 0.5 % methylcellulose solution (vehicle) at the volume of 1 ml/kg. The animals of group 2 received vehicle of 1 ml/kg. Another two groups of rats (3 and 4) receiving candesartan and vehicle, respectively, were similarly handled but not stressed. Next day, after ending the repeated stress procedure, all rats underwent behavioural testing.

### Behavioural tests

#### Inhibitory avoidance

Retrieval of an aversively motivated behaviour was tested by the IA paradigm (Ader et al. 1972). The test was conducted in a one-trial learning, step-through situation, which utilises the natural preference of rats for dark environment. After 2 min of habituation to the dark compartment, the rat was placed on the illuminated platform and allowed to enter into the dark compartment. Two more approach trials were given on the following day with a 2-min interval. At the end of the second trial, unavoidable scrambled electric footshock (0.25 mA, AC, 3 s) was delivered through the grid floor of the dark compartment (learning trial). The retrieval of IA was tested 24 h later by placing the animal on the platform and measuring the latency to re-enter into the dark compartment to a maximum of 300 s.

#### Object recognition

Memory of an appetitively (by curiosity) motivated behaviour was studied in an OR test (Ennaceur and Meliani 1992). The apparatus consisted of a plastic box 62-cm long, 38-cm wide and 20-cm high covered with a wire mesh lid. The objects to be discriminated were made of glass or porcelain and existed in duplicate. They appeared to have no natural significance for the rats, and they had never been associated with reinforcement. They were heavy enough not to be displaced by the rats. The procedure included two habituation sessions, with a 1-h interval, whereby the rats were allowed for 3-min exploration of the apparatus. Twenty-four hours later, the testing began. The experimental session consisted of two trials, each lasting 3 min. In the first trial (T1), the rats were exposed to two identical objects A. In the second trial (T2), performed 60 min later, the rats were exposed to two objects, one of which was a duplicate of the familiar object A (A′), in order to avoid olfactory traits, and a new object B. From rat to rat, the role (familiar or new object) as well as the relative position of the two objects were counterbalanced and randomly permuted during trial T2. These precautions were taken in order to reduce object and place preference effects. The basic measure was the time spent by the rat in exploring objects during trials T1 and T2. Exploration of an object was defined as touching it with the nose. Turning around or sitting on the object was not considered an exploratory behaviour. The following variables were defined: *A*, the time spent in exploring, objects A in T1; *A*′ and *B*, the times spent in exploring respectively, the duplicate of familiar and the new object in T2. Object recognition was measured by the variable *B* − *A*′, and total exploration in T2, by *B* + *A*′. Moreover, as *B* − *A′* may be biassed by the differences in the overall levels of exploration, the variable ${{{B - A \prime}} \left/ {{B + A \prime}} \right.}$ was also computed.

#### Open field

Locomotor exploratory activity was measured in an open field which was a square white floor measuring 100 × 100 cm divided by eight lines into 25 equal squares and surrounded by a 27-cm high wall as described earlier (Braszko et al. 1987)

#### Elevated plus maze

Anxiety was evaluated in an elevated plus maze (EPM) (constructed of grey-coloured wooden planks) consisting of two open arms, 50 cm (length) × 10 cm (width), and two enclosed arms 50 cm (length) × 10 cm (width) × 40 cm (height), covered by a removable lid, such that the open or closed arms were opposite to each other. The maze was elevated to a height of 50 cm from the floor. The rat was placed for 5 min in a pretest arena (60 × 60 × 35 cm, constructed of the same material) prior to exposure to the maze. This step allows facilitation of exploratory behaviour. The experimental procedure was similar to that originally described by Pellow et al. (1985) with some modifications (Braszko 2004).

### Experimental design

All behavioural experiments were performed the next day after ending the repeated stress procedure. Each group of animals underwent two kinds of tests provided that the results of the second test were unlikely to be biassed by the results of the first one. Accordingly, the rats tested for IA behaviour (a 3-day test) after a 2-min habituation trial performed at about 1000 hours on day 1 were then tested for locomotor exploratory activity at 1400 hours on the same day. The rats tested for OR in the morning were then tested for anxiety in the EPM in the afternoon the same day at the times as above.

Next day, after ending the experiments, the stressed rats were randomly checked for gastric ulceration. Every fifth animal was anaesthetized with the mixture of ketamine and diazepam (60:4.5 mg/kg i.p.) and sacrificed. Its stomach mucosa was then exposed, washed and examined visually under the × 5 magnification lens for gastric ulceration. In all cases, the mucosa appeared pink with no visible signs of injury of any kind.

### Statistical analysis

The result of all the experiments were evaluated by ANOVA I followed by Newman–Keuls test. Levels were deemed significant at *p* < 0.05.

## Results

### Effects of the repeated stress and chronic candesartan on retrieval of the inhibitory avoidance behaviour

**Fig. 1:**
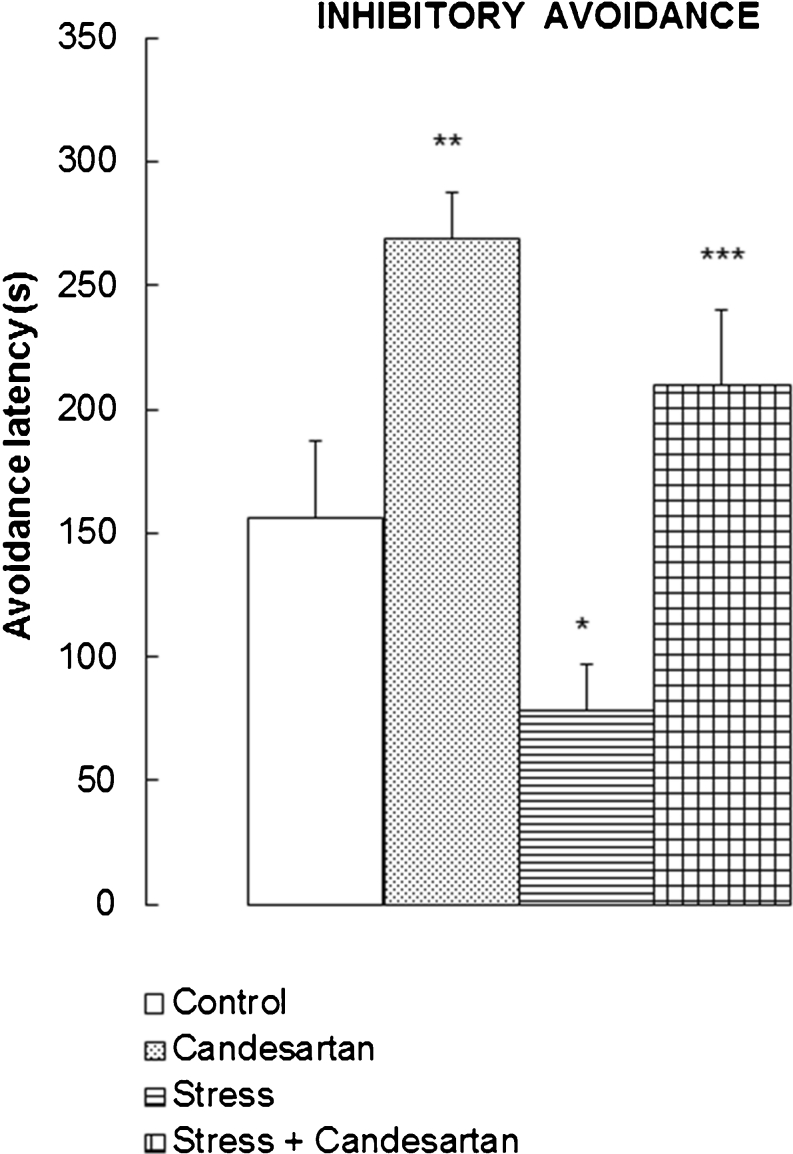
Effect of chronic candesartan (0.1 mg/kg/day, p.o., 21 days), chronic restraint stress (2 h/21 days) or both in combination, on the latency of the inhibitory avoidance. *Bars* represent means + SEM from 14–16 rats. ANOVA I and Newman–Keuls test: *p < 0.05, vs control; **p < 0.01, vs control and stress groups; ***p < 0.01, vs stress group

ANOVA I of the times spent by all the rats on illuminated platform of the apparatus yielded *F*(3,56) = 9.913, *p* < 0.0005, showing thus statistically significant group differences (Fig. 1). Pairwise comparisons between the groups made with Newman–Keuls test revealed the rats treated with candesartan to have statistically significantly (*p* < 0.01) better retrieval of memory of the IA behaviour than the control rats. The animals repeatedly stressed showed opposite; they had statistically significantly (*p* < 0.05) worse retrieval of memory of the IA behaviour in comparison to the control. The latter effect was abolished in the stressed rats chronically treated with candesartan. These animals had statistically significantly (*p* < 0.01) better retrieval of memory of IA than the stressed but not candesartan-treated rats.

### Effects of the repeated stress and chronic candesartan on object recognition memory

The times spent in exploring objects A (variable *A*) was comparable in all groups (Table 1). ANOVA I of the discrimination rates (*B* − *A*′) in all rats yielded *F*(3,56) = 14.954, *p* < 0.0001, showing thus statistically significant differences between the groups. Pairwise comparisons made with Newman–Keuls test showed that the object recognition memory (*B* − *A*′) was statistically significantly worse (*p* < 0.05) in the stressed group in comparison with the control group and this effect was reversed by candesartan. The stressed candesartan-treated animals had significantly (*p* < 0.05) better object recognition memory than the stressed but not treated rats. In addition, not stressed candesartan-treated animals also displayed statistically significantly (*p* < 0.001) better recognition memory than the control not stressed and not candesartan treated-rats. There were no statistically significant effects of our treatments on the remaining parameters measured in the OR test. Specifically, we observed no changes in habituation (*B* + *A*′) and no bias of the OR scores by the changes in the overall levels of exploration $(B - A\prime /B + A\prime )$

**Table 1:** Effect of chronic candesartan (cand) and chronic stress on object recognition

Variables (s)	Treatment			
	Control	Stress	Cand^a^	Stress + cand
A	31.87 ± 1.76^b^	35.28 ± 2.95	42.0 ± 3.63	38.86 ± 2.29
A′	40.37 ± 3.44	36.78 ± 4.49	28.87 ± 3.43	31.28 ± 3.61
B	42.94 ± 3.98	33.07 ± 3.49	47.44 ± 5.76	35.93 ± 4.43
B − A′	1.94 ± 1.87	−6.14 ± 1.80*	18.69 ± 4.038**	4.64 ± 2.03***
B + A′	82.69 ± 7.21	69.85 ± 7.58	76.31 ± 8.57	67.21 ± 7.81
$B - A\prime /B + A\prime$	0.023 ± 0.025	−0.1 ± 0.036	0.24 ± 0.087	0.069 ± 0.047

^a^Rats were given candesartan (0.1 mg/kg/day, p.o., 21 days) and chronically stressed. For further details, see text

^b^Values (in seconds) are means (± SEM)

^*^ *p* < 0.05; ^**^ *p* < 0.001 (vs control and stress + candesartan); ^***^ *p* < 0.05 (vs stress and vs candesartan; ANOVA I and Newman–Keuls test; for further details, see text)

### Effects of the repeated stress and chronic candesartan on the locomotor exploratory activity in the open field

**Fig. 2:**
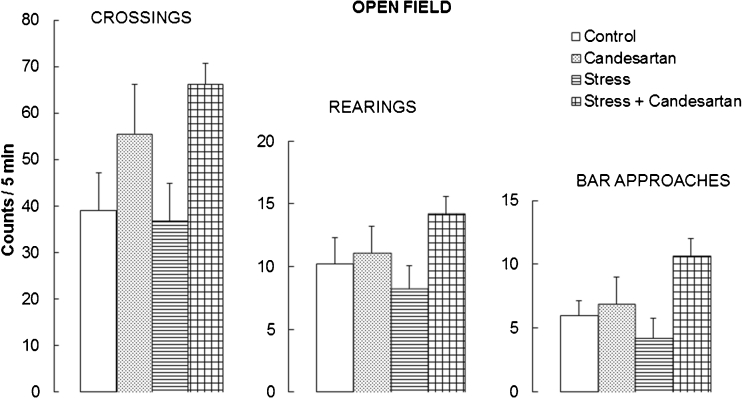
Effect of chronic candesartan (0.1 mg/kg/day, p.o., 21 days), chronic restraint stress (2 h/21 days) or both in combination, on the locomotor exploratory activity of rats in the open field. *Bars* represent means + SEM from nine rats

No statistically significant differences were found in the numbers of crossing, rearings and bar approaches counted in the open field in any of our experimental groups (Fig. 2). ANOVA I of the counts of these behavioural parameters yielded *F*(3,32) = 2.966, *F*(3,32) = 1.744 and *F*(3,32) = 2.994 (*p* > 0.05 in all cases) showing no statistically significant differences between the groups. This experiment demonstrates no appreciable influence of unspecific motor effects of the applied treatments on the results of our cognitive test.

### Effect of the repeated stress and chronic candesartan on the behaviour of rats in the elevated plus maze

**Fig. 3:**
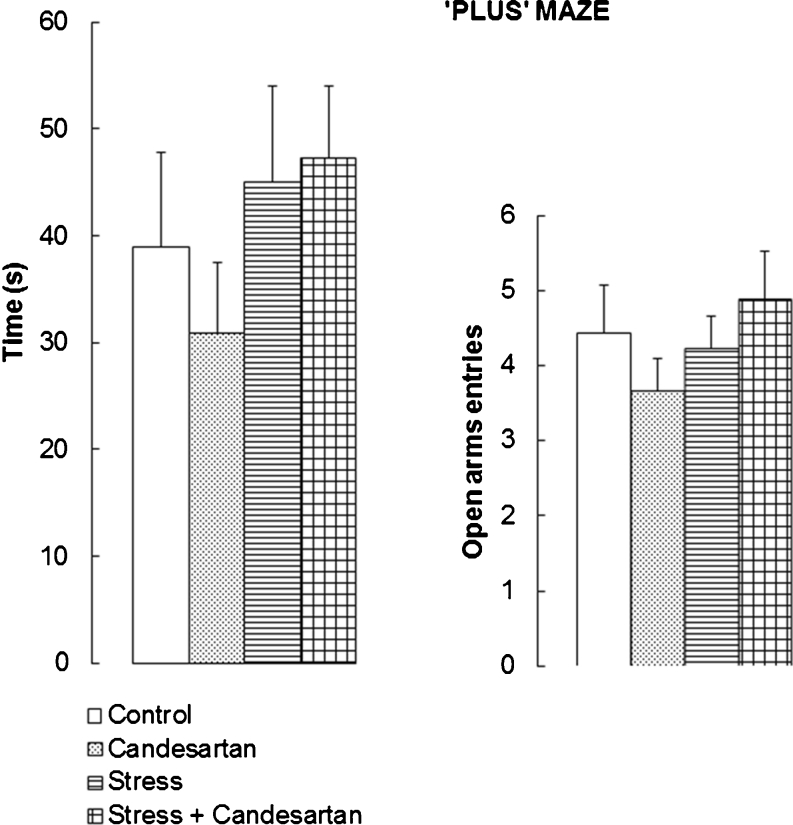
Effect of chronic candesartan (0.1 mg/kg/day, p.o., 21 days), chronic restraint stress (2 h/21 days) or both in combination on the time spent by rats in the open arms of the elevated “plus” maze and the number of entries therein. *Bars* represent means + SEM from nine rats

ANOVA I of the times spent by individual rats in the open arms of the elevated plus maze yielded *F*(3,32) = 0.857, *p* > 0.05, showing thus no significant differences between the groups (Fig. 3). Also, ANOVA I of numbers of the open arm entries made by individual rats yielded *F*(3,32) = 0.858, *p* > 0.05 pointing to absence of statistically significant differences between any of the four compared groups. Again, this experiment allowed the conclusion that a nonspecific influence of fear/anxiety on the results of our cognitive tests was unlikely.

## Discussion

This study demonstrated that candesartan, a highly selective AT_1_ Ang II receptor antagonist (Nishikawa et al. 1997), effectively prevented deleterious effects of the repeated stress on retrieval of memory in rats. To clearly dissect out the effect of candesartan on memory in both not stressed and repeatedly stressed animals, we had to ensure lack of influence of our stress procedure and candesartan on at least three factors: (1) locomotor activity, (2) anxiety and (3) blood pressure. These factors could obviously affect the animals' performance in our cognitive tests (Braszko et al. 2003) that would unspecifically bias their outcome. The results of the open field and the elevated plus maze tests excluded the first two factors, and the low nonhypotensive dose of candesartan (Nishikawa et al. 1997), purposefully used in this study, ensured lack of excessive lowering of blood pressure in our rats.

Although several chronic stress procedures have been shown to cause gastric ulceration (Salim 1988), the restraint paradigm of 2 h daily/21 days employed in the present study was harmless in this respect. An explanation might be in the strain of Wistar rats used in our experiments that is considered more resistant to stress-induced gastric ulceration in comparison to the other strains such as spontaneously hypertensive rats, Wistar–Kyoto or Sprague–Dawley Rats used in similar studies (Pare 1990). Altogether, in our model, a mild psychosocial stress (McLaughlin et al. 2007) causing no typical pathology such as gastric ulcers, decreased locomotion or anxiety appeared to be used. This was a fortunate situation as all these additional pathologies would make correct evaluation of the results of our cognitive tests difficult or even impossible. Interestingly however, a more severe, 6 h daily/21 days, stress procedure was used by other authors to produce cognitive deficits well correlated with hippocampal CA3 neuron dendrite retraction (McLaughlin et al. 2007).

Although involvement of the renin–angiotensin system in various stress-induced pathologies has been extensively studied for almost two decades (for review, see Saavedra et al. 2011), to our knowledge its participation in the impairment of memory caused by stress has not been specifically addressed. In face of the increasing number of stressful challenges brought by our daily life, the importance of this issue cannot be overestimated. Fortunately, a number of recently published data indicate, in addition to the present results, that the popular drugs being AT_1_ Ang II receptor inhibitors collectively called sartans (Saavedra et al. 2011) may offer at least partial solution of the problem. Sartans at present constitute the first-line treatment of hypertension (Sestito 2011), a condition being a main risk factor for myocardial infarction and stroke, the leading causes of overall mortality. The sartans introduced to clinic in 1993 (Sneader 2005) and increasingly used ever since (Sestito 2011) proved to be a safe and effective medication for hypertension and related conditions such as left ventricular hypertrophy and diabetic nephropathy (Chrysant 2008; Gansevoort et al. 1994; Ichihara et al. 2001). Chronic stress is a well-known causative factor of hypertension (Kvetnansky et al. 2009; Saavedra et al. 2011). Therefore, in all cases of hypertension in which stress is implicated as an aetiological factor, AT_1_ receptor blockade appears to be much better treatment choice than the other drugs. The mechanism of alleviation of hypertension-induced cognitive deficits by angiotensin receptor type 1 blockers (the sartans) most probably involves their ability to stimulate peroxisome proliferator-activated receptor gamma (PPAR-γ) (Fournier et al. 2009; Saxby et al. 2008; Schrader et al. 2007; Trenkwalder 2006). PPAR-γ agonism exerted mainly by telmisartan (Haraguchi et al. 2010; Mogi et al. 2008; Tsukuda et al. 2009) but to a different extent also by all other sartans including losartan, irbesartan and candesartan (Min et al. 2012; Schupp et al. 2004) was found to be responsible for the memory improvements after repeated cerebral ischemia in rats (Haraguchi et al. 2010) and β-amyloid injection (Tsukuda et al. 2009) or type 2 diabetes mellitus in mice (Min et al. 2012). Activation of the PPAR-γ is also considered to participate in antidiabetic, anti-inflammatory, and cholesterol profile-improving effects of several drugs (Saavedra et al. 2011). In line with these considerations is a recent clinical study demonstrating that sartans improve memory and delay progression of Alzheimer's disease, when compared to the other antihypertensive medications (Li et al. 2010).

Importantly, the recall-alleviating effect of candesartan was, in our present experiments, achieved at its very low dose (0.1 mg/kg) only slightly affecting blood pressure in rats (Nishikawa et al. 1997). Interestingly, candesartan was effective in protecting memory of both aversively motivated amygdala-dependent IA behaviour (Ehrlich et al. 2009) and appetitively, by curiosity, motivated medial prefrontal cortex-dependent (Nelson et al. 2011) OR, bringing in both cases retrieval of memory damaged by stress to normal. Again, memory of both behaviours which was impaired by a 21-day chronic restraint stressing procedure was fully preserved with low oral dose of candesartan. For explanation of this preventive, against chronic stress-induced cognitive impairment, action of candesartan blockade to most if not all of the adverse effects of excessive AT_1_ receptor signalling including overexpression of the AT_1_ receptors in PVN (Saavedra et al. 2006), decreased production of CRH by parvocellular part of PVN and increased pituitary ACTH content causing increased adrenal corticosterone production (Armando et al. 2001, 2007; Baiardi et al. 2004) leading, among other effects, to hippocampal atrophy (Herbert et al. 2006; McLaughlin et al. 2007) appears most probable. In addition, corticosterone (cortisol in humans) further increases AT_1_ receptor expression in all parts of the HPA axis creating thus a self-perpetuating mechanism (Saavedra et al. 2004) leading to an allostatic overload (McEwen 2005; McEwen and Stellar 1993), i.e. energy-consuming, exhausting for an organism, new homeostasis (allostasis).

AT_1_ receptor blockade appears to break this sequence of events at several levels including the brain cortex, hippocampus, amygdala, hypothalamus, pituitary and adrenal glands (Saavedra et al. 2011). Detailed mechanisms of translation of this blockade to prevention of several stress-induced pathologies including gastric ulceration, anxiety, posttraumatic stress disorder, depression, hypertension and memory disturbances are at present unknown and warrant further research.

Interestingly, candesartan clearly improved recall of the IA behaviour not only in the stressed but also in not stressed animals. Although explanation of this memory-enhancing effect of the drug is difficult, alleviation of certain level of the basal anxiety present in control rats may be considered. Candesartan might then improve IA performance as the result of emotional disinhibition. Such a normal cognition-improving effect of another anti-stress medication with Hypericum perforatum extracts was previously described by Khalifa (2001) and in our recent study (Trofimiuk and Braszko 2008).

Anxiety-like behaviour in the plus maze was usually (Bondi et al. 2008; Lapiz-Bluhm et al. 2008) but not always (Cunningham et al. 2009) observed as a result of a chronic restraint with additional unpredictable stressing stimuli, such as an unscheduled lighting, wet bedding, unexpected noise, etc. in Sprague–Dawley rats. Our 21-day/2-h daily stress procedure in Wistar rats, conducted without the additional stress stimuli, has never produced significant alterations in the parameters of anxiety measured in the plus maze (Walesiuk et al. 2005; Trofimiuk et al. 2005; Trofimiuk and Braszko 2011).

In conclusion, these results present experimental evidence that the impairment of memory retrieval caused by chronic restraint stress can be fully prevented by low concomitantly administered dose of candesartan. The drug is already widely used as an antihypertensive (Khawaja and Wilcox 2011) and clinical trials designed to test its, and possibly other sartans, efficacy in stress-related memory deficits should, in our opinion, be conducted.

## References

[CR1] Ader R, Weijnen JAWM, Moleman P (1972). Retention of a passive avoidance responses as function of the intensity and duration of electric shock. Psychon Sci.

[CR2] Aguilera G, Kiss A, Luo X (1995). Increased expression of type 1 angiotensin II receptors in the hypothalamic paraventricular nucleus following stress and glucocorticoid administration. J Neuroendocrinol.

[CR3] Aguilera G, Young WS, Kiss A, Batahia A (1995). Direct regulation of hypothalamic corticotrophin-releasing-hormone neurons by angiotensin II. Neuroendocrinology.

[CR4] Armando I, Carranza A, Nishimura Y, Hoe KL, Barontini M, Terron JA, Falcon-Neri A, Ito T, Juorio AV, Saavedra JM (2001). Peripheral administration of angiotensin II AT_1_ receptor antagonist decreases the hypothalamic-pituitary-adrenal response to isolation stress. Endocrinology.

[CR5] Armando I, Volpi S, Aguilera G, Saavedra JM (2007). Angiotensin II AT_1_ receptor blockade prevents the hypothalamic corticotrophin-releasing factor response to isolation stress. Brain Res.

[CR6] Baiardi G, Bregonzio C, Jezova M, Armando I, Saavedra JM (2004). Angiotensin II AT_1_ receptor blockade prolongs the lifespan of spontaneously hypertensive rats and reduces stress-induced release of catecholamines, glucocorticoids, and vasopressin. Ann NY Acad Sci.

[CR7] Bondi CO, Rodriguez G, Gould GG, Frazer A, Morilak DA (2008). Chronic unpredictable stress induces a cognitive deficit and anxiety-like behavior in rats that is prevented by chronic antidepressant drug treatment. Neuropsychopharmacology.

[CR8] Braszko JJ (2004). Involvement of D_1_ dopamine receptors in the cognitive effects of angiotensin IV and des-Phe^6^ angiotensin IV. Peptides.

[CR9] Braszko JJ, Wiśniewski K, Kupryszewski G, Witczuk B (1987). Psychotropic effects of angiotensin II and III in rats: locomotor and exploratory vs. cognitive behavior. Behav Brain Res.

[CR10] Braszko JJ, Kułakowska A, Winnicka MM (2003). Effects of angiotensin II and its receptor antagonists on motor activity and anxiety in rats. J Physiol Pharmacol.

[CR11] Buchanan TW, Tranel D, Adolphs R (2006). Impaired memory retrieval correlates with individual differences in cortisol response but not autonomic response. Learn Mem.

[CR12] Castren E, Saavedra JM (1988). Repeated stress increases the density of angiotensin II binding sites in the rat paraventricular nucleus and subfornical organ. Endocrinology.

[CR13] Chrysant SG (2008). Angiotensin II receptor blockers in the treatment of the cardiovascular disease continuum. Clin Ther 30Pt.

[CR14] Cunningham JI, Raudensky J, Tonkiss J, Yamamoto B (2009). MDMA Pretreatment leads to mild chronic unpredictable stress-induced impairments in spatial learning. Behav Neurosci.

[CR15] deQuervain DJ, Roozendaal B, McGauhg JL (1998). Stress and glucocorticosteroids impair retrieval of long-term spatial memory. Nature.

[CR16] deQuervain DJ, Roozendaal B, Nitsch RM, McGauch JL, Hock C (2000). Acute cortisone administration impairs retrieval of long-term declarative memory in humans. Nat Neurosci.

[CR17] deQuervain AJ-F, Aerni A, Schelling G, Roozendal B (2009). Glucocorticoids and the regulation of memory in health and disease. Front Neuroendocrinol.

[CR18] Ehrlich I, Humeau Y, Grenier F, Ciocchi S, Herry C, Lűthi A (2009). Amygdala inhibitory circus and the control of fear memory. Neuron.

[CR19] Ennaceur A, Meliani K (1992). A new one-trial test for neurobiological studies of memory in rats. III. Spatial vs. nonspatial working memory. Behav Brain Res.

[CR20] Fournier A, Oprisiu-Fournier R, Serot JM, Godefroy O, Achard JM, Faure S, Mazouz H, Temmar M, Albu A, Bordet R, Hanon O, Gueyffier F, Wang J, Black S, Sato N (2009). Prevention of dementia by antihypertensive drugs: how AT1-receptor-blockers and dihydropyridines better prevent dementia in hypertensive patients than thiazides and ACE-inhibitors. Expert Rev Neurother.

[CR21] Ganong WF, Murakami K (1987). The role of angiotensin in the regulation of ACTH secretion. Ann NY Acad Sci.

[CR22] Gansevoort RT, De Zeeuw D, Shahinfar S, Redfirld A, De Jong PE (1994). Effects of angiotensin II antagonist losartan in hypertensive patients with renal disease. J Hypertens.

[CR23] Haraguchi T, Iwasaki K, Takasaki K, Uchida K, Naito T, Nogami A, Kubota K, Shindo T, Uchida N, Katsurabayashi S, Mishima K, Nishimura R, Fujiwara M (2010). Telmisartan, a partial agonist of peroxisome proliferator-activated receptor γ, improves impairment of spatial memory and hippocampal apoptosis in rats treated with repeated cerebral ischemia. Brain Res.

[CR24] Herbert J, Goodyer IM, Grossman AB, Hastings MH, de Kloet ER, Lightman SL, Lupien SJ, Roozendal B, Seckl JR (2006). Do corticosteroids damage the brain?. J Neuroendocrinol.

[CR25] Ichihara S, Senbonmatsu T, Price E, Ichiki T, Gaffney FA, Inagami T (2001). Angiotensin II type 2 receptor is essential for left ventricular hypertrophy and cardiac fibrosis in chronic angiotensin II-induced hypertension. Circulation.

[CR26] Jezova D, Ochedalski T, Kiss A, Aguilera G (1998). Brain angiotensin II modulates sympathoadrenal and hypothalamic pituitary adrenocortical activation during stress. J Neuroendocrinol.

[CR27] Keller-Wood M, Kimura B, Shinsako J, Philips MI (1986). Interaction between CRF and angiotensin II in control of ACTH and adrenal steroids. Am J Physiol.

[CR28] Khalifa AE (2001). *Hypericum perforatum* as a nootropic drug: enhancement of retrieval memory of a passive avoidance conditioning paradigm in mice. J Etnopharmacol.

[CR29] Khawaja Z, Wilcox CS (2011). An overview of candesartan in clinical practice. Expert Rev Cardiovasc Ther.

[CR30] Kvetnansky R, Sabban EL, Palkovits M (2009). Catecholaminergic systems in stress: structural and molecular genetic approaches. Physiol Rev.

[CR31] Lapiz-Bluhm MDS, Bondi CO, Doyen J, Rodriguez G, Bedard-Arana T, Morilak DA (2008). Behavioral assays to model cognitive and affective dimensions of depression and anxiety in rats. J Neuroendocrinol.

[CR32] Leong DS, Terron JA, Falcon-Neri A, Armando I, Ito T, Johren O (2002). Restraint stress modulates brain, pituitary and adrenal expression of angiotensin II AT_1A_ and AT_1B_ and AT_2_ receptors. Neuroendocrinology.

[CR33] Li NC, Lee A, Whitmer RA, Kivipelto M, Lawler E, Kazis LE, Wolozin B (2010). Use of angiotensin receptor blockers and risk of dementia in a predominantly male population: prospective cohort analysis. Br Med J.

[CR34] Magarinos AM, Verdugo JMG, McEwen BS (1997). Chronic stress alters synaptic terminal structure in hippocampus. Proc Natl Acad Sci USA.

[CR35] McEwen BS (2005). Glucocorticoids, depression, and mood disorders: structural remodeling in the brain. Metab Clin Exp.

[CR36] McEwen BS, Stellar E (1993). Mechanisms leading to disease. Arch Intern Med.

[CR37] McLaughlin KJ, Gomez JL, Baran SE, Conrad CD (2007). The effects of chronic stress on hippocampal morphology and function: an evaluation of chronic restraint paradigms. Brain Res.

[CR38] Min LJ, Mogi M, Shudou M, Jing F, Tsukuda K, Ohshima K, Iwanami J, Horiuchi M (2012). Peroxisome proliferator-activation with angiotensin II type 1 receptor blockade is pivotal for the prevention of blood-brain barrier impairment and cognitive decline in type 2 diabetic mice. Hypertension.

[CR39] Mogi M, Li JM, Tsukuda K, Iwanami J, Min LJ, Sakata A, Fujita T, Iwai M, Horiuchi M (2008). Telmisartan prevented cognitive decline partly due to PPAR-gamma activation. Biochem Biophys Res Commun.

[CR40] Nelson AJD, Cooper MT, Thur KE, Marsden CA, Cassady HJ (2011). The effect of catecholaminergic depletion within the prelimbic and infralimbic medial prefrontal cortex on recognition memory for recency, location, and object. Behav Neurosci.

[CR41] Nishikawa K, Naka T, Chatani F, Yoshimura Y (1997). Candesartan cilexetil: a review of its preclinical pharmacology. J Hum Hypertens.

[CR42] Pare WP (1990). Technique and strain comparisons in stress ulcer. Ann NY Acad Sci.

[CR43] Pellow S, Chopin P, Briley M (1985). Validation of open: closed arm entries in an elevated plus-maze as a measure of anxiety in the rat. J Neurosci Meth.

[CR44] Saavedra JM (2005). Brain angiotensin II: new developments, unanswered questions and therapeutic opportunities. Cell Mol Neurobiol.

[CR45] Saavedra JM, Ando H, Armando I, Baiardi G, Bregonzio C, Jezova M, Zhou J (2004). Brain angiotensin II, an important stress hormone: regulatory sites and therapeutic opportunities. Ann NY Acad Sci.

[CR46] Saavedra JM, Armando I, Breginzio C, Juorio A, Macova M, Pavel J, Sanchez-Lemus E (2006). A centrally acting, anxiolytic angiotensin II AT_1_ receptor antagonist prevents the isolation stress-induced decrease in cortical CRF_1_ receptor and benzodiazepine binding. Neuropsychopharmacology.

[CR47] Saavedra JM, Sanchez-Lemus E, Benicky J (2011). Blockade of brain angiotensin II AT_1_ receptors ameliorates stress, anxiety, brain inflammation and ischemia: therapeutic implications. Psychoneuroendocrinology.

[CR48] Salim AS (1988). The hypothalamus and gastric mucosal injuries: origin of stress-induced injury?. J Psychiatr Res.

[CR49] Saxby BK, Harrington F, Wesnes KA, McKeith IG, Ford GA (2008). Candesartan and cognitive decline in older patients with hypertension: a substudy of the SCOPE trial. Neurology.

[CR50] Schrader J, Kulschewski A, Dendorfer A (2007). Inhibition of the rennin-angiotensin system and the prevention of stroke. Am J Cardiovasc Drugs.

[CR51] Schupp M, Janke J, Clasen R, Unger T, Kintscher U (2004). Angiotensin type 1 receptor blockers induce peroxisome proliferator-activated receptor-gamma activity. Circulation.

[CR52] Sestito A (2011). Hypertension therapy and cardiovascular protection. Effects of angiotensin II receptor block with valsartan. Eur Rev Med Pharmacol Sci.

[CR53] Sneader W (2005). Drug discovery: a history.

[CR54] Sumimoto T, Suda T, Nakano Y, Tozawa F, Yamada M, Demura H (1991). Angiotensin II increases the corticotrophin-releasing factor messenger ribonucleic acid levels in the rat hypothalamus. Endocrinology.

[CR55] Terfehr K, Wolf OT, Schlosser N, Carvahlo Fernando S, Otte C, Muhtz C, Beblo T, Driessen M, Spitzer C, Löwe B, Wingenfeld K (2011). Hydrocortisone impairs working memory in healthy humans, but not in patients with major depressive disorder. Psychopharmacology.

[CR56] Trenkwalder P (2006). The study on cognition and prognosis in the elderly (SCOPE)—recent analyses. J Hypertens Suppl.

[CR57] Trofimiuk E, Braszko JJ (2008). Alleviation by *Hypericum perforatum* of the stress-induced impairment of spatial working memory in rats. Naunyn-Schmied Arch Pharmacol.

[CR58] Trofimiuk E, Braszko JJ (2011). Long-term administration of cod liver oil ameliorates cognitive impairment induced by chronic stress in rats. Lipids.

[CR59] Trofimiuk E, Walesiuk A, Braszko JJ (2005). St John's wort (*Hypericum perforatum*) diminishes cognitive impairment caused by the chronic restraint stress in rats. Pharmacol Res.

[CR60] Trofimiuk E, Hołownia A, Braszko JJ (2011). St. John's wort may relieve negative effects of stress on spatial working memory by changing synaptic plasticity. Naunyn-Schmied Arch Pharmacol.

[CR61] Tsukuda K, Mogi M, Iwanami J, Min L-J, Sakata A, Jing F, Iwai M, Horiuchi M (2009). Cognitive deficit in amyloid-β-injected mice was improved by pretreatment with a low dose of telmisartan partly because of peroxisome proliferator-activated receptor-γ activation. Hypertension.

[CR62] Walesiuk A, Trofimuik E, Braszko JJ (2005). Ginkgo biloba extract diminishes stress-induced memory deficits in rats. Pharmacol Rep.

[CR63] Walesiuk A, Trofimiuk E, Braszko JJ (2006). Ginkgo biloba normalizes stress- and corticosterone-induced impairment of recall in rats. Pharmacol Res.

[CR64] Wolf OT (2009). Stress and memory in humans: twelve years of progress?. Brain Res.

[CR65] Woodson JC, Macintosh D, Fleshner M, Diamond DM (2003). Emotion-induced amnesia in rats: working memory-specific impairment, corticosterone-memory correlation, and fear versus arousal effects on memory. Learn Mem.

[CR66] Xang G, Xi ZX, WAN Y, Wang H, Bi G (1993). Changes in circulating and tissue angiotensin II during acute and chronic stress. Biol Signals.

[CR67] Yang G, Wan Y, Zhu Y (1996). Angiotensin II—an important stress hormone. Biol Signals.

